# Functional Neuroimaging Correlates of Aggression in Psychosis: A Systematic Review With Recommendations for Future Research

**DOI:** 10.3389/fpsyt.2018.00777

**Published:** 2019-02-05

**Authors:** Sonja Widmayer, Stefan Borgwardt, Undine E. Lang, Rolf-Dieter Stieglitz, Christian G. Huber

**Affiliations:** ^1^Department of Psychiatry, University Hospital Basel, University of Basel, Basel, Switzerland; ^2^Psychological Faculty, University of Basel, Basel, Switzerland

**Keywords:** aggression, psychosis, schizophrenia, functional magnetic resonance imaging, systematic review

## Abstract

**Background and methods:** Aggression in psychosis is clinically important. We systematically compiled the evidence on functional correlates of aggression in psychosis searching PubMed, EMBASE, ScienceDirect, and PsycINFO until September 2017. We included studies reporting functional brain imaging correlates of aggression comparing: (1) affective or non-affective psychosis groups with a history of violence or with aggression operationalized using questionnaires, (2) affective or non-affective psychosis groups with a history of violence or with aggression operationalized using questionnaires to controls, (3) affective or non-affective psychosis groups with a history of violence or with aggression operationalized using questionnaires to controls with diagnoses other than affective or non-affective psychoses. We applied no language restriction and required patients to have a DSM or ICD diagnosis of affective or non-affective psychosis.

**Results:** Our sample consisted of 12 studies with 334 patients and 113 controls. During n-back tasks, violent (VS) as opposed to non-violent persons with schizophrenia (NVS) hypoactivated their inferior parietal lobe. When anticipating shock, VS vs. NVS hyperactivated their medial prefrontal gyrus, cuneus, middle temporal gyrus, and middle occipital gyrus. When viewing negative emotional pictures, VS vs. NVS hyperactivated the middle frontal gyrus, inferior frontal gyrus, anterior cingulate, lingual gyrus, precentral gyrus, globus pallidus, mid-cingulate, and precuneus.

**Limitations:** Due to the small number of available studies, sample overlap, and insufficient reporting of relevant moderators we could not conduct a meta-analysis.

**Conclusions:** We found non-systematic functional correlates of aggression in schizophrenia. Only few studies using varied paradigms and often overlapping samples have been conducted. There have been no attempts to replicate any of the observed findings in the published literature. Focusing on future directions, we recommend that authors adhere to clear definitions of aggression, measurements of psychopathology, comorbidities, and medication. In particular, replication studies would allow for a better synthesis of the findings.

**PROSPERO Registration Number:** CRD42016048579

## Introduction

Most persons with schizophrenia are not violent (1)– less than 10% of violent crimes in society is attributable to schizophrenia (2). According to Brekke et al. (3), persons with schizophrenia are at highly increased risks of becoming victims of violence.

Still, some of the patients suffering schizophrenia are at increased risks for aggressive behavior (4–6), even in the first episode of illness (7–10), and in at-risk mental states (11). This poses severe challenges to the patients themselves, their families and health care professionals. In this systematic review, we pooled the available evidence on functional neuroimaging correlates of aggression in psychosis—of which there is surprisingly little—to provide an overview on the biological underpinnings of this clinically important phenomenon and give advice for the future development of the field.

### Functional Neuroimaging Findings in Aggression

#### Functional Neuroimaging Correlates of Aggression in Healthy Persons

Lotze et al. (12) found hyperactivated medial prefrontal cortices in healthy controls (HC) reacting aggressively in a game paradigm during fMRI. The dorsomedial PFC was hyperactivated when subjects selected the intensity of the aggressive response.

Pietrini et al. (13), in a PET study, found that HC hypoactivated their ventromedial prefrontal cortices in aggressive vs. neutral scenarios.

An important particularity implicated in aggression seems to be the hyperactivity of the limbic system in response to negative or provocative stimuli, particularly anger provoking stimuli (14).

#### Functional Neuroimaging Correlates of Aggression in Patient Populations

Persons with impulsive aggression and HC underwent fMRI while viewing emotional faces (15). Relative to controls, patients exhibited higher amygdala and lower orbitofrontal cortex (OFC) activation to angry faces. While HC did, aggressive subjects did not couple amygdala-OFC when seeing angry faces.

New et al. (16) provoked aggression in a laboratory setting with the Point Subtraction Aggression Paradigm. Patients with borderline personality disorder with an anger dyscontrol, as opposed to HC, showed hypoactivations to provocation in the medial frontal cortex and the anterior frontal cortex but hyperactivations in the orbital frontal cortex.

### Preliminary Work on Functional Neuroimaging Correlates of Aggression in Psychosis

Some reviews have compiled data on functional neuroimaging correlates of aggression in psychoses.

Naudts and Hodgins (17) suggest that people with schizophrenia with as opposed to those without violent behavior perform better in executive function tasks and show less impairments in the dorsolateral prefrontal cortex. Hoptman and Antonius (18) sum up that frontal and temporal particularities seem to be a consistent feature of aggression in schizophrenia but that their nature remains unclear. Soyka (19) concludes that in schizophrenia patients with aggression as opposed to those without aggression certain brain functions may be more severely impaired.

Existing reviews have been conducted 6 years ago and are narrative expert reviews lacking systematic literature search and quantitative statistical measures. As to our knowledge, there are currently no studies reporting fMRI correlates of aggression in persons with affective psychoses.

### Research Question

We aimed at conducting a systematic literature research and compiling all available data on functional neuroimaging correlates of aggression in patients with psychotic disorders: Are there systematic differences in the brain activation patterns in aggressive vs. non-aggressive persons with psychotic disorders? What recommendations for future research can be proposed based on the current state of the literature?

## Methods

We adopted the “Preferred Reporting Items for Systematic Reviews and Meta-Analyses” (PRISMA) guidelines (20) and the revised “Quality of Reporting of Meta-analyses” QUORUM statements (21). The study protocol was registered on the International Prospective Register of Systematic Reviews database (PROSPERO; registration number: CRD42016048579) prior to the completion of data extraction. To assess the methodological quality of the current systematic review, we used the PRISMA 2009 checklist (20).

### Search and Selection Strategy

We conducted a systematic review of fMRI studies on correlates of aggression in patients with psychoses vs. HC. We searched the PubMed, EMBASE, ScienceDirect, and PsycINFO databases with no restriction on start date until September 2017. We used search thesauri representing aggression, psychosis and functional brain imaging. The detailed search terms can be found on http://www.crd.york.ac.uk/PROSPERO/display_record.asp?ID=CRD42016048579. Furthermore, we searched the reference lists of all included original articles for additional literature.

Subsequently, we screened all studies according to the following inclusion criteria. We included longitudinal, cross-sectional, and case-control studies (journal articles, book chapters, and dissertations) reporting brain imaging correlates of aggression comparing: (1) affective or non-affective psychosis groups with a history of violence or including continuous measures of aggression, (2) affective or non-affective psychosis groups with a history of violence or including continuous measures of aggression to HC, (3) affective or non-affective psychosis groups with a history of violence or including continuous measures of aggression to controls with diagnoses other than affective or non-affective psychoses. Furthermore, we included all studies with brain imaging using functional MRI and with an age of cases/controls of at least 18 years. We applied no language restriction and required patients to have an established diagnosis of affective or non-affective psychosis according to DSM or ICD.

If there was insufficient information to extract data, we contacted corresponding authors by email using coding sheets to collect study data. When we received no response from the authors, we contacted them again—then, if we received no response, we excluded the study.

#### Quality Assessment

The quality of the studies was assessed using an item-checklist constructed specifically for the current work and similar to the previously published quality assessment by Paulson and Bazemore (22). The recorded variables were assessed in terms of precision, directness and consistency of the data. The categories scored in the quality assessment are listed in Supplementary Table 1 (0–2 points per item with a theoretical range of 0–38 for the total quality score). The included studies were rated according to the sum of the points and characterized as high quality (≥80% of the maximal sum of points), moderate-high (60–79%), moderate (40–59%), moderate-low (20–39%), and low quality studies (<20%). Six of the included studies had a high quality, four had a moderate-high quality, one had moderate quality, and one study moderate-low quality.

### Data Extraction

The main outcome measures were the hyper- and hypoactivations in the specific brain regions of patient groups [violent persons with schizophrenia (VS) and non-violent persons with schizophrenia (NVS)] and HC. We extracted imaging center, name of the first author, year of publication, type of functional imaging analysis, population characteristics of HC and patient groups (group size, gender, age, IQ, violence score, psychopathology, medication), operationalization of aggression, stimulation material, sum of points in quality assessment of individual studies, and diagnosis from all studies.

### Data Analysis

We performed a qualitative analysis of all included publications. Talairach coordinates were transformed into MNI coordinates using GingerALE software, version 2.3; available from http://brainmap.org/ale/ (23, 24). To provide a clearer overview on hyper- and hypoactivation patterns in the different tasks and groups over all papers using similar paradigms, we produced multislice activation pattern figures (**Figures 2**–**5**) in MRIcron (25) using the reported MNI coordinates for building three dimensional ROIs (version from 2nd of May 2016; available from http://people.cas.sc.edu/rorden/mricron/install.html).

## Results

### Literature Search

**Figure 1:**
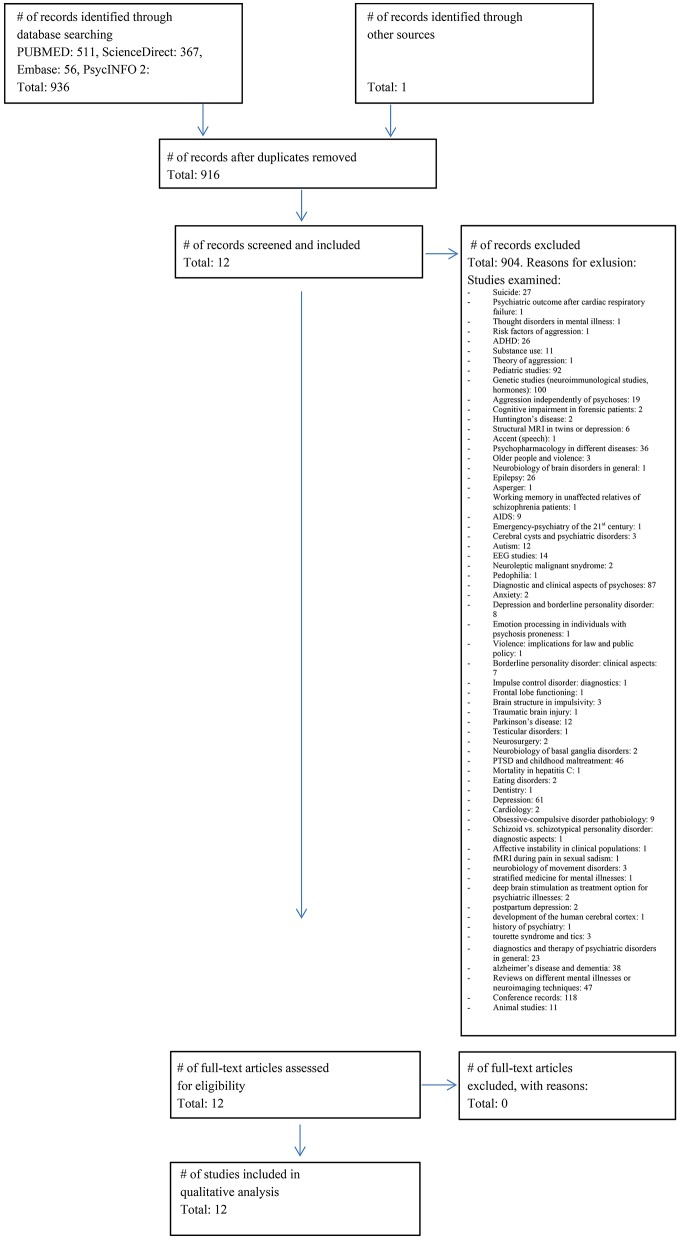
Prisma flow chart. Flowchart of the literature search (03.09.2017) and included studies according to the PRISMA guidelines (20).

The literature search identified 937 studies of interest. After screening and applying in- and exclusion criteria, 925 studies were excluded. Main reasons for these numerous exclusions were that included persons did not fulfill the relevant diagnostic criteria or that the paper did not examine correlates of aggression. The exact number of studies with specific reasons for exclusion are detailed in Figure 1.

Using the PRISMA template, we summarize the study selection procedure in Figure 1.

The final sample consisted of 12 studies with a total of 334 patients and 113 HC. After subtracting subject overlaps due to the publication of multiple papers using the same cohort, the sample consisted of 236 patients and 92 HC subjects. Barkataki et al. (26), Kumari et al. (27), and Kumari et al. (28) as well as both Wong et al. (29) and Wong et al. (30) used the same underlying patient population.

Diagnoses were either schizophrenia or schizoaffective disorder according to DSM-IV or ICD-10—no studies included affective psychoses.

Table 1 gives an overview of all included studies showing imaging center; name of the first author; year of publication; type of functional imaging analysis; population characteristics of HC, and patient groups (group size, gender, age, IQ, violence score, psychopathology, medication); operationalization of aggression; stimulation material; sum of points in quality assessment of individual studies; diagnosis.

**Table 1:**
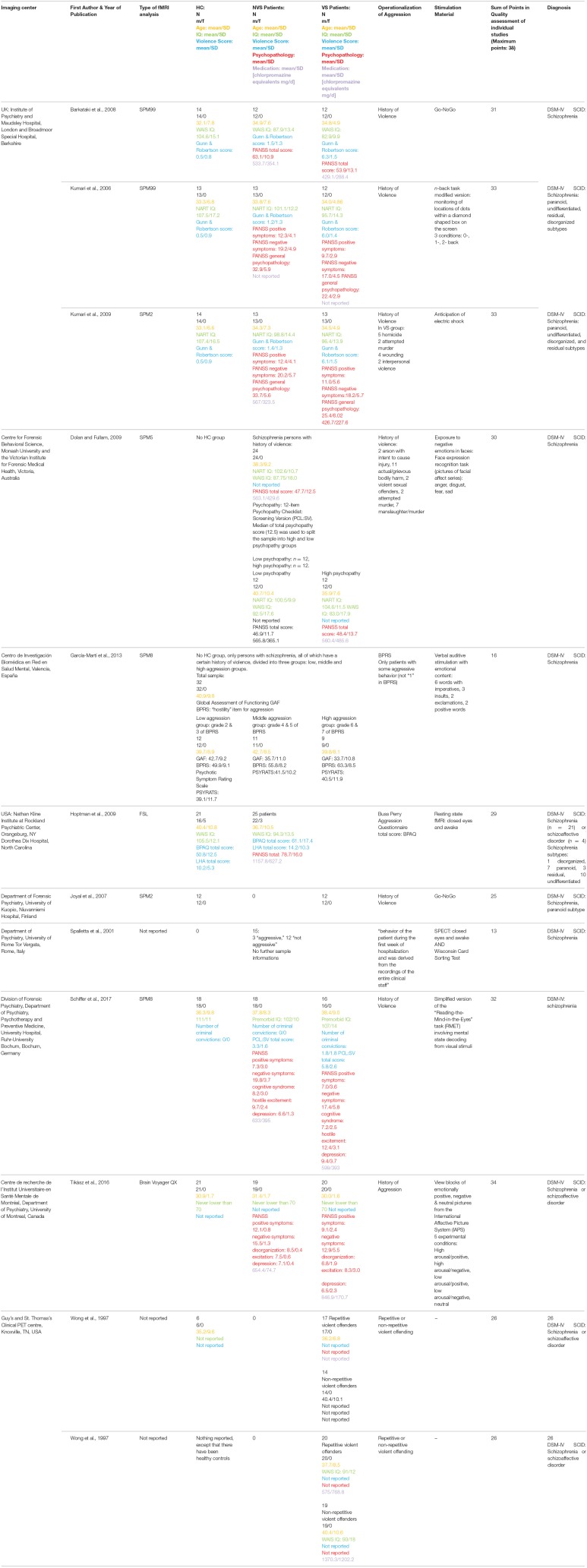
Overview of included studies.

*Overview of the studies included in the qualitative report describing imaging center; name of the first author; year of publication; type of imaging analysis; population characteristics of healthy controls and patient groups; operationalization of aggression; stimulation material; quality score; diagnosis. HC $\overset{\wedge }{=}$ healthy controls, NVS $\overset{\wedge }{=}$ non-violent schizophrenia group, VS $\overset{\wedge }{=}$ violent schizophrenia group, HoV $\overset{\wedge }{=}$ History of Violence, PANSS $\overset{\wedge }{=}$ Positive and Negative Syndrome Scale, NART IQ $\overset{\wedge }{=}$ National Adult Reading Test for estimating premorbid intelligence levels, WAIS IQ $\overset{\wedge }{=}$ Wechsler Adult Intelligence Scale to measure cognitive ability in adults, Gunn & Robertson Scale $\overset{\wedge }{=}$ 5-item scale for rating violence and other criminal activities, DSM-IV $\overset{\wedge }{=}$ Diagnostic and Statistical Manual of Mental Disorders, fourth edition, SCID $\overset{\wedge }{=}$ Structured Clinical Interview for DSM disorders*.

### Activation Patterns

Table 2 shows fMRI activation patterns across all tasks, brain areas, and group comparisons. For readers interested in more detail in the individual studies, the following sections summarize their results and provide tables about the specific areas and tasks involved. Bearing in mind Table 2, readers can choose to jump directly to the discussion section.

**Table 2:**
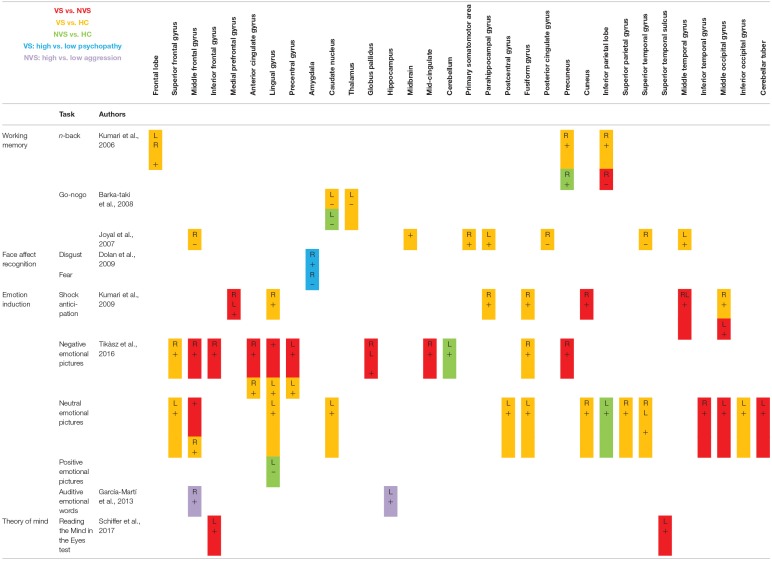
Activation pattern overview.

*Activation patterns in different tasks over all studies. +, Hyperactivation; –, Hypoactivation; L, left; R, right*.

### Working Memory

When operationalizing working memory functioning with Go/No-Go, VS vs. HC hypoactivated the right middle frontal gyrus, right posterior cingulate gyrus, and right superior temporal gyrus (31). In contrast, VS as opposed to HC hyperactivated the left middle temporal gyrus, midbrain, right primary somatomotor area, left parahippocampal gyrus (31) and hypoactivated the left thalamus and left caudate nucleus (26). NVS as opposed to HC hypoactivated solely the left caudate nucleus.

Working memory as measured by the n-back task showed that VS vs. HC hyperactivated the frontal lobe bilaterally, the right precuneus, and the right inferior parietal lobe. NVS vs. HC hyperactivated the right precuneus, while VS vs. NVS hypoactivated the right inferior parietal lobe (27). Table 3 shows an overview.

**Table 3:**
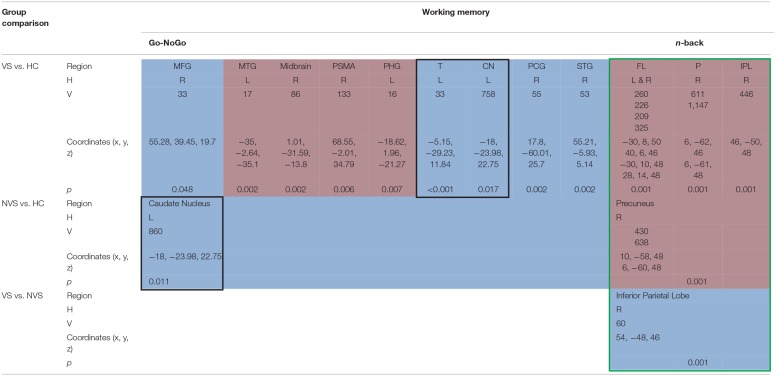
Activation patterns in working memory.

*Hyperactivations and Hypoactivations. H $\overset{\wedge }{=}$ Hemisphere, V $\overset{\wedge }{=}$ Cluster Size in Voxels, Coordinates ? MNI. MFG $\overset{\wedge }{=}$ Middle Frontal Gyrus, MTG $\overset{\wedge }{=}$ Middle Temporal Gyrus, PSMA $\overset{\wedge }{=}$Primary Somatomotor Area, PHG $\overset{\wedge }{=}$ Parahippocampal Gyrus, T $\overset{\wedge }{=}$ Thalamus, CN $\overset{\wedge }{=}$ Caudate Nucleus, PCG $\overset{\wedge }{=}$ Posterior Cingulate Gyrus, STG $\overset{\wedge }{=}$ Superior Temporal Gyrus, FL $\overset{\wedge }{=}$ Frontal Lobe, P $\overset{\wedge }{=}$ Precuneus, IPL $\overset{\wedge }{=}$ Inferior Parietal Lobe. Black box $\overset{\wedge }{=}$ Results reported by Barkataki et al. (26), green box $\overset{\wedge }{=}$ results reported by Kumari et al. (27). Results without box are reported by Joyal et al. (31)*.

### Face Affect Recognition

Dolan and Fullam (32) compared VS with high vs. low psychopathy using a face affect recognition task. When viewing a disgustful face, high psychopathy VS hyperactivated the right amygdala—when viewing a fearful face, they hypoactivated it (see Table 4).

**Table 4:** Activation patterns in face affect recognition.

**Group comparison**		**Face affect recognition**	
		**Disgust**	**Fear**
VS: high vs. low psychopathy	Region	Amygdala	Amygdala
	H	R	R
	V	3	2
	Coordinates (x, y, z)	28, −4, −21	25, 0, −21
	*p*	0.026	0.026

*Hyperactivations and Hypoactivations as reported by (33). H $\overset{\wedge }{=}$ Hemisphere, V $\overset{\wedge }{=}$ Cluster Size in Voxels, Coordinates $\overset{\wedge }{=}$ MNI*.

### Emotion Induction

#### Shock Anticipation

Kumari et al. (28) conducted a fMRI study where participants (VS, NVS and HC) were threatened to receive an electric shock. Under shock anticipation, VS as opposed to HC hyperactivated the right fusiform/inferior temporal gyrus as well as the right lingual/posterior cingulate gyrus.

VS vs. NVS hyperactivated the medial prefrontal/cingulate gyrus bilaterally, middle temporal gyrus bilaterally, right posterior cingulate/cuneus, and left middle occipital gyrus (see Table 5A).

**Table 5A:** Activation patterns in emotion induction: shock anticipation.

**Group comparison**				**Emotion induction**			
				**Shock anticipation**			
VS vs. HC	Region	FITG	LPCG				
	H	R	R				
	V	1,168	4,527				
	Coordinates (x, y, z)	42, −52, −16 28, −44, −6 30, −58, −12	24, −66, 2 28, −58, −2 26, −74, 20				
	*p*	0.031	<0.001				
VS vs. NVS	Region			MPCG	MTG	PCC	MOG
	H			R L L	R L	R	L
	V			2,176	4,008 1,155	Not reported	1,256
	Coordinates (x, y, z)			20, 32, −14 −6, 24, −2 −16, 26, −8	42, −60, 16 50, −12, −2 −44, −34, −14 −36, −26, −6	26, −74, 14	−28, −84, 10 −32, −76, 24 −20, −66, 6
	*p*			<0.001	<0.001 0.016	Not reported	0.01

*Hyperactivations and Hypoactivations as reported by Kumari et al. (28). H $\overset{\wedge }{=}$ Hemisphere, V $\overset{\wedge }{=}$ Cluster Size in Voxels, Coordinates $\overset{\wedge }{=}$ MNI. FITG $\overset{\wedge }{=}$ Fusiform/Inferior Temporal Gyrus, LPCG $\overset{\wedge }{=}$ Lingual/Posterior Cingulate Gyrus, MPCG $\overset{\wedge }{=}$ Medial Prefrontal/Cingulate Gyrus, MTG $\overset{\wedge }{=}$ Middle Temporal Gyrus, PCC $\overset{\wedge }{=}$ Posterior Cingulate/Cuneus, MOG $\overset{\wedge }{=}$ Middle Occipital Gyrus*.

#### Emotional Pictures

Tikàsz et al. (34) showed negative emotional pictures to VS, NVS, and HC and observed functional alterations in brain activity (see Table 5B).

**Table 5B:** Activation patterns in emotion induction: negative emotional pictures.

**Group comparison**		**Emotion induction**														
		**Negative emotional pictures**														
VS vs. HC	Region	FG	SFG	AC	LG	PG										
	H	R	R	R	L	L										
	V	12,498	362	1,031	1,852	1,389										
	Coordinates (x, y, z)	40.12, −76.88, −6.63	17.64, 46.08, 35.38	40.15, 48.68, 4.99	−18.32, −84.4, −14.97	−40.62, 3.78, 33.83										
	*p*	<0.001	<0.001	<0.001	<0.001	<0.001										
NVS vs. HC	Region						STG	C	PCG	H	IPL					
	H						R L	L	L	L	L					
	V						402 765	1,619 614	6,264	1,905	2,579					
	Coordinates (x, y, z)						36.22, 16.55, −39.35 −50.3, −51.75, 26.04	−5.86, −42.18, −46.26 −31.7, −54.72, −41.21	−1.87, −36.56, 13.63	−34.6, −32.47, −9.75	−43.48, −49.23, 52.57					
	*p*						<0.001	<0.001	<0.001	<0.001	<0.001					
VS vs. NVS	Region			AC	LG	PG						MFG	IFG/STG	GP	P	MC
	H			R	R	L						R	R	R L	R	R
	V			2,159	98,791	1,828						1,217	903	1,027 2,052	728	1,027
	Coordinates (x, y, z)			4.1, 41.68, −11.02	1.36, −89.15, 1.97	−43.89, 0.26, 30.87						36.61, 58.99, 0.16	36.51, 8.95, 18.42	26.92, −16.3, 12.4 −21.67, −12.97, −8.54	24.54, −72.26, 43.61	4.73, 0.43, 30.03
	*p*			<0.001	<0.001	<0.001						<0.001	<0.001	<0.001	<0.001	<0.001

*Negative Emotional Pictures. Hyperactivations and Hypoactivations as reported by Tikàsz et al. (34). H $\overset{\wedge }{=}$ Hemisphere, V $\overset{\wedge }{=}$ Cluster Size in Voxels, Coordinates $\overset{\wedge }{=}$ MNI. FG $\overset{\wedge }{=}$ Fusiform Gyrus, SFG $\overset{\wedge }{=}$ Superior Frontal Gyrus, AC $\overset{\wedge }{=}$ Anterior Cingulate, LG $\overset{\wedge }{=}$ Lingual Gyrus, PG $\overset{\wedge }{=}$ Precentral Gyrus, STG $\overset{\wedge }{=}$ Superior Temporal Gyrus, C $\overset{\wedge }{=}$ Cerebellum, PCG $\overset{\wedge }{=}$ Posterior Cingulate Gyrus, H $\overset{\wedge }{=}$ Hippocampus, IPL $\overset{\wedge }{=}$ Inferior Parietal Lobe, MFG $\overset{\wedge }{=}$ Middle Frontal Gyrus, IFG/STG $\overset{\wedge }{=}$ Inferior Frontal Gyrus/Superior Temporal Gyrus, GP $\overset{\wedge }{=}$ Globus Pallidus, P $\overset{\wedge }{=}$ Precuneus, MC $\overset{\wedge }{=}$ Mid–Cingulate*.

VS vs. HC hyperactivated the right fusiform gyrus, right superior frontal gyrus, right anterior cingulate, left lingual gyrus, and left precentral gyrus.

NVS vs. HC hyperactivated the superior temporal gyrus bilaterally, left cerebellum, left posterior cingulate gyrus, left hippocampus, and left inferior parietal lobe.

VS vs. NVS hyperactivated right anterior cingulate, right lingual gyrus, left precentral gyrus, right middle frontal gyrus, right inferior frontal gyrus, and superior temporal gyrus, globus pallidus bilaterally, right precuneus, and right mid-cingulate.

Tikàsz et al. (34) showed also positive and neutral emotional pictures to their participants (see Table 5C).

**Table 5C:** Activation patterns in emotion induction: positive and neutral emotional pictures.

**Group comparison**	**Emotion induction**															
		**Positive emotional pictures**	**Neutral emotional pictures**													
VS vs. HC	Region		MFG	STG	SPG	C	CN	SFG	PG	LG	IOG	FG	IPG	ITG	MOG	CT
	H		R	R L	R	R	L	L	L	L	L	L				
	V		15,541	904 1,393	2,987	1,080	5,679	1,209	14,975	1,031	8,061	408				
	Coordinates (x, y, z)		66.33, 17.23, 34.04	46.02, 17.31, −32.86 −41.39, 4.55, −26.74	34.31–52.15, 51.54	24.32, −93.59, 22.19	−15.26, 19.27, −8.47	−2.06, 67.1, 20.19	−49.97, −26.3, 57.14	−11.87, 87.88, −18.1	−50.55, −86.46, −0.77	−24.85, 71.66, −16.12				
	*p*		<0.001	<0.001	<0.001	<0.001	<0.001	<0.001	<0.001	<0.001	<0.001	<0.001				
NVS vs. HC	Region	LG											IPG			
	H	L											L			
	V	571											1007			
	Coordinates (x, y, z)	−15.23, −33.02, −16.75											−36.99, −42.51, 55.16			
	*p*	< 0.001											<0.001			
VS vs. NVS	Region		MFG											ITG	MOG	CT
	H		R											R	L	L
	V		355											462	42,793	496
	Coordinates (x, y, z)		0.26, 46.49, −28.25											62.49, −49.64, −26.51	−37.46, 85.48, 8.99	−60.74, −53.89, −30.72
	*p*		<0.001											<0.001	<0.001	<0.001

*Neutral Emotional Pictures. Hyperactivations and Hypoactivations as reported by Tikàsz et al. (34). H $\overset{\wedge }{=}$ Hemisphere, V $\overset{\wedge }{=}$ Cluster Size in Voxels, Coordinates $\overset{\wedge }{=}$ MNI. MFG $\overset{\wedge }{=}$ Middle Frontal Gyrus, STG $\overset{\wedge }{=}$ Superior Temporal Gyrus, SPG $\overset{\wedge }{=}$ Superior Parietal Gyrus, C $\overset{\wedge }{=}$ Cuneus, CN $\overset{\wedge }{=}$ Caudate Nucleus, SFG $\overset{\wedge }{=}$ Superior Frontal Gyrus, PG $\overset{\wedge }{=}$ Postcentral Gyrus, LG $\overset{\wedge }{=}$ Lingual Gyrus, IOG $\overset{\wedge }{=}$ Inferior Occipital Gyrus, FG $\overset{\wedge }{=}$ Fusiform Gyrus, IPG $\overset{\wedge }{=}$ Inferior Parietal Gyrus, ITG $\overset{\wedge }{=}$ Inferior Temporal Gyrus, MOG $\overset{\wedge }{=}$ Middle Occipital Gyrus, CT $\overset{\wedge }{=}$ Cerebellar Tuber*.

When viewing positive pictures, NVS vs. HC hypoactivated the left lingual gyrus.

VS vs. HC showed significant hyperactivations in response to neutral pictures in right middle frontal gyrus, right superior temporal gyrus, right superior parietal gyrus, right cuneus, left caudate nucleus, left superior frontal gyrus, left postcentral gyrus, left lingual gyrus, left inferior occipital gyrus, and left fusiform gyrus. NVS vs. HC hyperactivated the left inferior parietal gyrus.VS vs. NVS, when viewing neutral pictures, hyperactivated right middle frontal gyrus, right inferior temporal gyrus, left middle occipital gyrus, and left cerebellar tuber.

#### Emotional Words

García-Martí et al. (35) stimulated their subjects with auditive emotional words. In this paradigm, NVS with high aggression scores as opposed to those with low aggression scores hyperactivated the left hippocampus and the right medial frontal cortex (see Table 5D).

**Table 5D:** Activation patterns in emotion induction: auditive emotional words.

**Group comparison**		**Auditive emotional words**	
NVS: high vs. low aggression	Region	Hippocampus	Medial Frontal Cortex
	H	L	R
	V	6.078 cm^3^	13.251 cm^3^
	Coordinates (x, y, z)	−36, −14, 10	31, 52, 18
	p	0.003	0.004

*Auditive Emotional Words. Hyperactivations and Hypoactivations as reported by García-Marti et al. (35). H $\overset{\wedge }{=}$ Hemisphere, V $\overset{\wedge }{=}$ Cluster Size in Voxels, Coordinates $\overset{\wedge }{=}$ MNI*.

### Affective Theory of Mind

Schiffer et al. (36) showed a picture of a person's eyes to their participants and let them select—out of two words—the one that fitted best the person's emotional state. They found that, when performing this task, VS as compared to NVS hypoactivated the left ventrolateral PFC and left superior temporal sulcus at the temporoparietal junction (see Table 6).

**Table 6:** Activation patterns in theory of mind task: reading the mind in the eyes test.

**Group comparison**		**Reading the mind in the eyes**	
VS vs. NVS	Region	STS	Ventrolateral PFC
	H	L	L
	V	257	286
	Coordinates (x, y, z)	−34, −70, 28	−36, 42, −4
	*p*	<0.05	<0.05

*Theory of Mind. Hyperactivations and Hypoactivations as reported by Schiffer et al. (36). H $\overset{\wedge }{=}$ Hemisphere, V $\overset{\wedge }{=}$ Cluster Size in Voxels, Coordinates $\overset{\wedge }{=}$ MNI, STS $\overset{\wedge }{=}$ superior temporal sulcus at temporoparietal junction, PFC $\overset{\wedge }{=}$ prefrontal cortex*.

### Activation Patterns as Described by Other Studies

Hoptman et al. (37) conducted a resting-state fMRI study to examine amygdala/ventral prefrontal cortex connectivity in aggression and schizophrenia with HC and patients with schizophrenia and schizoaffective disorder. Lower functional connectivity between amygdala and ventral PFC was associated with higher levels of self-rated aggression, life history of aggression and total arrests leading to the hypothesis that amygdala and ventral PFC functional connectivity may be compromised in aggressive schizophrenia (37).

Spalletta et al. (38) assessed the relationship between prefrontal function and aggression in schizophrenia using single photon emission tomography and evaluated patients at rest and during activation with the Wisconsin Card Sorting Test (WCST). Clinical staff recorded aggression ratings. Aggressive and non-aggressive patients did not differ in RCBF during rest. During WCST activation, aggressive vs. non-aggressive subjects hypoactivated the right middle and inferior PFC. The authors hypothesized that prefrontal dysfunction results in the loss of intellectual flexibility impairing social skills that are essential for non-aggressive solutions.

A PET study with VS showed that repetitive violent offenders hypoactivated the left anterior inferior temporal cortex, while non-repetitive violent offenders hypoactivated this region bilaterally (29). A similar population showed generalized hypometabolism. The authors conclude that anterior temporal structures may control aggression (30).

## Discussion

We found non-systematic functional correlates of aggression in schizophrenia. Despite the clinical importance of the topic, only a limited number of studies could be included in this first systematic review on functional neuroimaging correlates of aggression in persons with psychotic disorders. No original research data was available on persons with affective psychoses. Considering the 12 studies in patients with schizophrenia-spectrum disorders (SSD) that could be included, there was considerable sample overlap, and functional neuroimaging paradigms covered a broad range of tasks. While this enables a better overview over which mechanisms could play a role for aggression in SSD, it complicates an integration of the individual findings into a common framework and impedes a quantitative synthesis of the published results. Furthermore, all of the reported findings have yet to be successfully replicated or refuted.

### Working Memory

Working memory is known to be impaired in patients with schizophrenia and there have been reports that limited working memory capacity and functioning are predictive of a more impulsive decision-making style (39). In the following, we report functional magnetic resonance imaging correlates of working memory associated with aggressive as opposed to non-aggressive persons with schizophrenia.

#### Go/No-Go

Joyal et al. (31) compared go/no-go activation in VS with HC and found hypoactivations in the right middle frontal gyrus, right posterior cingulate gyrus, and right superior temporal gyrus but hyperactivations in the left middle temporal gyrus, right midbrain, right primary somatomotor area, and left parahippocampal gyrus. These results match with the hypothesis by Naudts and Hodgins (17), that VS might suffer from neural dysfunction affecting basal or orbital parts of the prefrontal cortex.

VS vs. HC hypoactivated the left thalamus and left caudate nucleus (26). The finding of decreased thalamus activation does not concord with a previous study where Manoach et al. (40) found that schizophrenia patients activated the thalamus during working memory, while HC did not.

NVS vs. HC hypoactivated the left caudate nucleus (26), a region crucial to executive functioning and working memory deficits in schizophrenia (41, 42). Caudate nucleus hypoactivation in VS vs. HC might therefore not be specific for aggression but rather for schizophrenia itself.

#### N-Back

Kumari et al. (27) found that VS vs. HC showed hyperactivations in the frontal lobe bilaterally, in the right precuneus and in the right inferior parietal lobe. When comparing NVS with HC, the authors observed hyperactivations in the right precuneus. These findings do not match with a previous study where patients with schizophrenia as opposed to HC showed hyperactivation of the dorsolateral prefrontal cortex, the inferior parietal cortex, and the anterior cingulate (43). Also, schizophrenia patients have been shown to hypoactivate their dorsolateral PFC and hyperactivate their ventrolateral PFC (44).

Furthermore, Kumari et al. (27) reported reduced right inferior parietal activity in the VS group compared to the NVS group and a strong negative association between right inferior parietal activity and the ratings of violence in both groups.

The frontal deficit in VS was only evident when compared to HC but not vs. NVS. These results might suggest non-significant hypoactivation in the frontal regions in NVS vs. HC. A deficit in the inferior parietal region affects executive functioning in schizophrenia and might be associated with violence (45). Furthermore, various studies show that a frontal dysfunction may be associated with violence (46).

#### Synthesis of Findings From Studies Using Working Memory Paradigms

**Figure 2:**
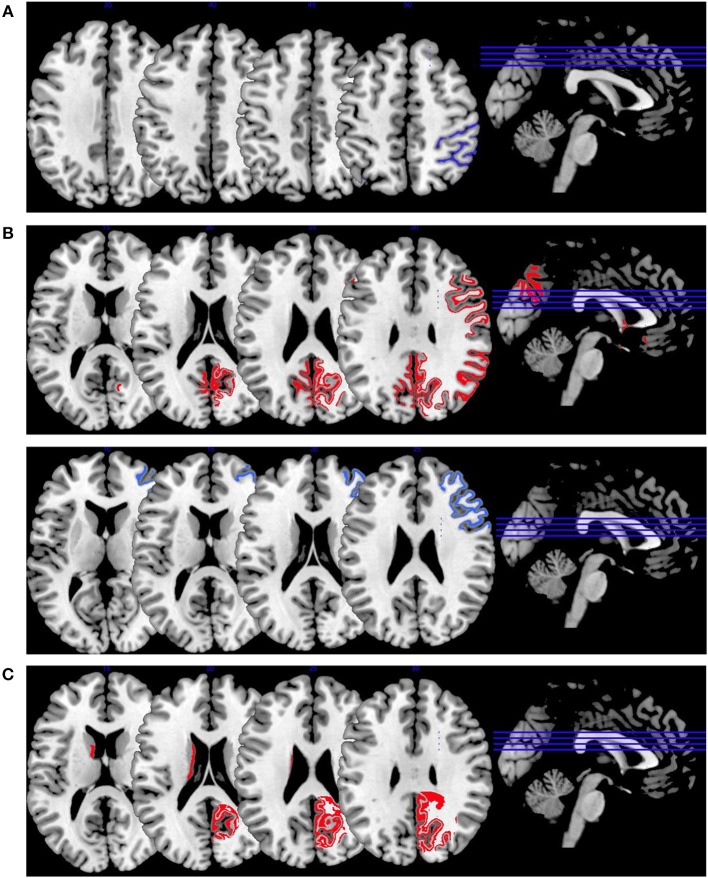
Working memory activations. Overview of the working memory brain activation patterns reported by the reviewed studies. Hyperactivations are shown in red, hypoactivations in blue. **(A)** shows the activation patterns in the group comparison VS vs. NVS with VS<NVS in blue (shown slice numbers are 210, 220, 230, 240) in the contrast 2-back vs. 0-back, **(B)** shows the activation patterns in the group comparison VS vs. HC with VS>HC in red (shown slice numbers are 170, 180, 190, 200) in the contrasts 1- and 2-back vs. 0-back and cognitive (go/no-go task) vs. reference condition and VS<HC in blue (shown slice numbers are 160, 170, 180, 190) in the contrast NoGo20 vs. NoGo40 and NoGo40 vs. Go and cognitive (go/no-go task) vs. reference condition, **(C)** shows the activation patterns in the group comparison NVS vs. HC with NVS>HC in red (shown slice numbers are 170, 180, 190, 200) in the contrast 1- and 2-back vs. 0-back.

In Figure 2, we provide an overview on working memory activation patterns over all reviewed studies. As shown in Figure 2A, violent as opposed to non-violent persons with schizophrenia showed hypoactivation in the right inferior parietal lobe. This is an area known for being part of the working memory network, but it has until now not been observed in specific aggression paradigms. The hypoactivation seen in this group comparison may therefore not be specific for aggression, but may represent working memory dysfunction in the VS group.

Figure 2B shows hyperactivations of VS as compared to HC mainly in the frontal lobe and in the middle temporal gyrus. In addition, we see hypoactivations of VS as opposed to HC in the right middle frontal gyrus, the cingulate gyrus, and in the superior temporal gyrus. As frontal regions are typically involved in working memory tasks, this finding is in line with the literature.

In Figure 2C, we present the activation patterns of NVS as compared to HC. NVS hyperactivate the left caudate nucleus and precuneus. The precuneus is known to be involved in working memory processes, while the caudate nucleus usually is not. Still, the latter—as a feedback processor—might be under higher workload conditions while solving these tasks in persons with schizophrenia than in healthy controls. Also, this could be an indicator toward the hypothesis that schizophrenia patients solve working memory tasks differently, namely trying to use information from past experiences to influence their decisions in the tasks.

### Face Affect Recognition

Individuals with schizophrenia suffer from difficulties in recognizing emotional states (47). Persons with antisocial behavior also tend to show deficits in recognizing facial emotion expression with the amygdala being specifically involved in the processing of fearful facial affect (48). In the following, we will in detail disentangle the results of studies on face affect recognition in persons suffering from schizophrenia with vs. without aggression.

VS with high psychopathy scores hyperactivated right amygdala when viewing and identifying disgustful faces, but hypoactivated it when viewing and identifying fearful faces (32). HC activate their left inferior frontal gyrus when viewing fearful faces (49). A meta-analysis by Fusar-Poli et al. (50) integrated findings stating that healthy controls activated the amygdala when viewing happy, fearful, and sad faces. Angry and disgusted faces activated the insula (50).

Persons with schizophrenia react to emotional stimuli by exhibiting reduced activation in the amygdala but increased activation in other regions that are usually not associated with emotion (51). Literature reporting brain activation to facial expressions in psychopathic samples is sparse. However, Deeley et al. (52) examined brain function as individuals with psychopathy and healthy controls processed facial emotion. When viewing happy faces, both groups hyperactivated the fusiform and extrastriate cortices, but this increase was significantly smaller in the psychopathy group. When processing fearful faces, healthy controls hyperactivated their fusiform gyrus while the psychopathy group hypoactivated it (52). These findings are not in line with the reviewed findings by Dolan and Fullam (32)—due to many influencing factors like e.g., mere effect of disease, level of psychopathy, and others it is very difficult to interpret these differential activation patterns. One study reported no neural dysfunction in response to angry faces in children with conduct disorder (53). There are no reports of impairments in the recognition of anger in psychopathic samples (48) or in patients with schizophrenia and high psychopathy scores (54). One study reported reduced arousal ratings in response to angry faces in psychopathic women (55). Neural responses are modulated by anxiety (56) and psychopathic traits are associated with low anxiety levels (57). High psychopathy scorers exhibited increased amygdala response to disgustful faces. There are no published studies on disgust in psychopaths but there are reports that individual differences in disgust sensitivity moderate neural responses to disgust stimuli in HC (58, 59).

#### Synthesis of Findings From Studies Using Face Affect Recognition Paradigms

**Figure 3:**
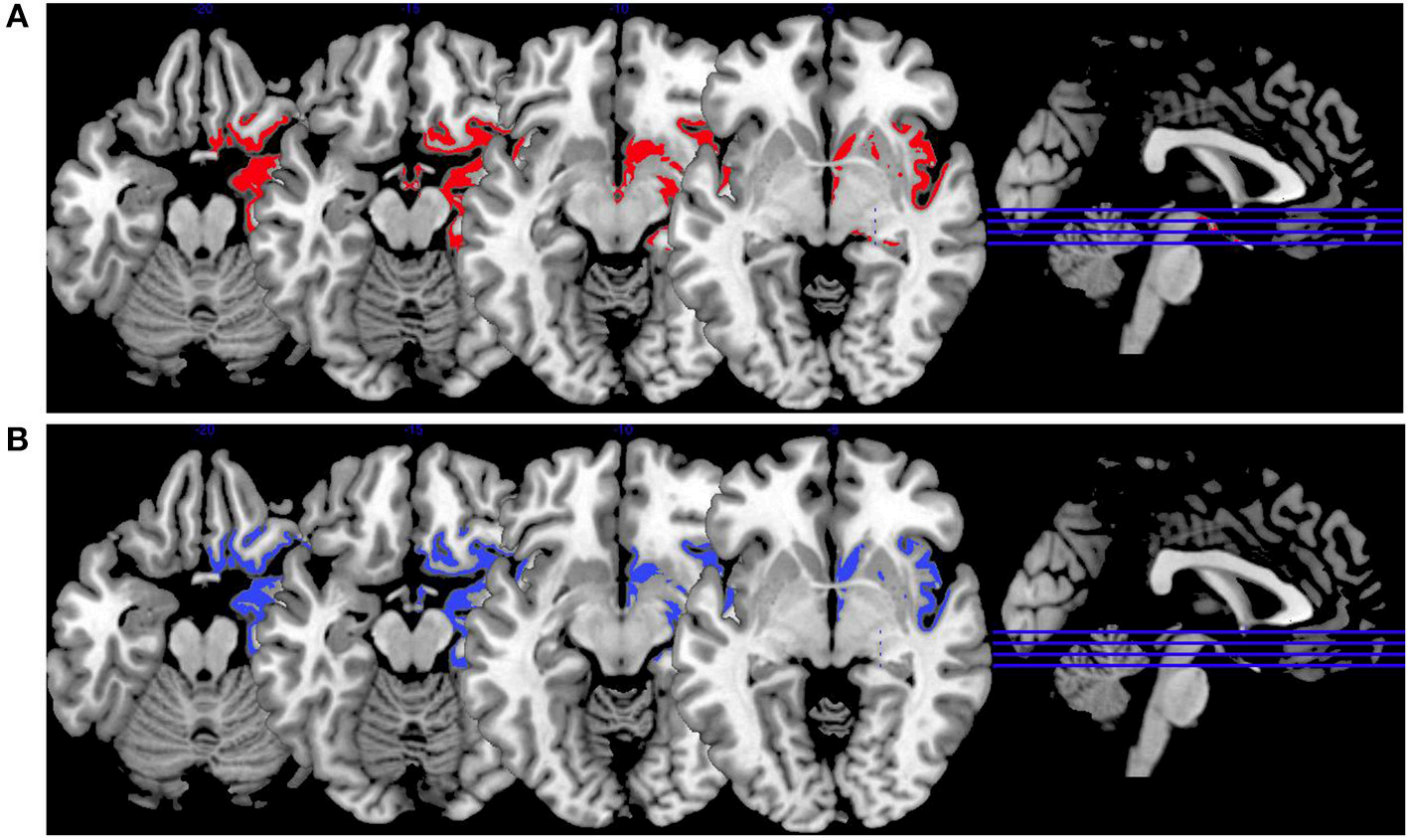
Face affect recognition activations. Overview of the working memory brain activation patterns reported by the reviewed studies in the group comparison VS high vs. VS low. Hyperactivations are shown in red, hypoactivations in blue. **(A)** Shows the activation patterns in the group comparison VS high vs. VS low with VS high>VS low in red showing the contrast of neutral vs. disgustful faces, **(B)** shows the activation patterns in the group comparison VS high vs. VS low with VS high<VS low in blue showing the contrast of neutral vs. fearful faces. Shown slice numbers are 100, 110, 120, 130.

In Figure 3, we provide an overview on activation patterns during face affect recognition over all reviewed studies. In Figure 3A, VS show hyperactivation in the right amygdala in persons with high as opposed to low psychopathy scores when viewing facial expressions of disgust. To our knowledge, there are no previous studies on brain activation patterns of aggressive persons on disgustful faces, but it has been observed that more aggressive persons exhibit higher amygdala activation when seeing angry faces (15).

When viewing fearful faces, as shown in Figure 3B, VS with high vs. low psychopathy hypoactivated the right amgydala. This is in line with findings reporting that persons with schizophrenia show hypoactivations of the amygdala in response to emotional stimuli.

### Emotion Induction

#### Shock Anticipation

Kumari et al. (28) found that when anticipating electric shock, violent persons with schizophrenia as opposed to HC show hyperactivations in right fusiform/inferior temporal gyrus as well as in right lingual/posterior cingulate gyrus.

When comparing violent with non-violent persons with schizophrenia under shock anticipation, violent persons with schizophrenia hyperactivated their medial prefrontal/cingulate gyrus bilaterally, middle temporal gyrus bilaterally, right posterior cingulate/cuneus, and left middle occipital gyrus.

Concluding, the AC, medial/inferior frontal regions, insula, striatum, and temporal regions were activated during anticipatory fear. These results are in line with a previous study by Chua et al. (60). The cingulate cortex and the insula are activated during emotional recall/imagery (61), while the neural representation of fear has been hypothesized to be located in the AC/medial prefrontal cortex (62).

#### Negative Emotional Pictures

Tikàsz et al. (34) compared violent schizophrenia with HC and observed significant hyperactivations as response to negative emotional pictures in the right fusiform gyrus, right superior frontal gyrus, right anterior cingulate, left lingual gyrus, and left precentral gyrus.

When comparing non-violent persons with schizophrenia with HC, non-violent persons with schizophrenia showed significantly higher activation in superior temporal gyrus bilaterally, left cerebellum, left posterior cingulate gyrus, left hippocampus, and left inferior parietal lobe. A previous meta-analysis had found no differences in amygdala activations between HC and persons with schizophrenia when exposed to aversive emotional stimuli (63).

Violent as opposed to non-violent persons with schizophrenia showed hyperactivations in response to negative emotional pictures in the right anterior cingulate, right lingual gyrus, left precentral gyrus, right middle frontal gyrus, right inferior frontal gyrus and superior temporal gyrus, globus pallidus bilaterally, right precuneus, and right mid-cingulate.

Tikàsz et al. (34) discussed the increase of the anterior cingulate reactivity to negative stimuli in the violent schizophrenia group as the most important finding. Some authors suggest that the anterior cingulate is crucial for the integration of negative affect and cognitive control (64–66). It is also associated with the generation and regulation of emotion (67) and, due to connections of the anterior cingulate with both the amygdala and the orbitofrontal cortex, the anterior cingulate seems to be critically involved in violent behavior (68). Tikàsz et al. (34) conclude that anterior cingulate dysfunctions are associated with negative stimuli processing in violent persons with schizophrenia and reason that negative emotions may be a factor in preceding violent behavior.

#### Positive and Neutral Emotional Pictures

Previous literature concerning healthy persons has shown that visual stimuli generally activate the occipital cortex and the amygdala (61). Furthermore, the medial prefrontal cortex was found to play a role in emotional processing across all categories independently of the emotion. Literature on neutral image processing in schizophrenia is sparse.

Non-violent persons with schizophrenia showed significantly lower activation in the left lingual gyrus than HC when viewing positive pictures.

In the neutral emotional pictures condition, violent persons with schizophrenia compared with HC showed significant hyperactivations in right middle frontal gyrus, right superior temporal gyrus, right superior parietal gyrus, right cuneus, left caudate nucleus, left superior frontal gyrus, left postcentral gyrus, left lingual gyrus, left inferior occipital gyrus, and left fusiform gyrus (34).

Non-violent persons with schizophrenia as opposed to HC showed significant hyperactivation in left inferior parietal gyrus (34). This finding fits into a previous study where Habel et al. (69) showed that persons with schizophrenia elicited hyperactivations in the frontal and cingulate areas and the basal ganglia.

Violent vs. non-violent persons with schizophrenia showed, when viewing neutral pictures, hyperactivations in the right middle frontal gyrus, right inferior temporal gyrus, left middle occipital gyrus, and left cerebellar tuber (34). The authors discuss that it remains unclear how these activation patterns are related to aggressive behavior.

#### Auditive Emotional Words

Sanjuan et al. (70) used an auditory paradigm to induce emotion presenting schizophrenia patients and HC with neutral and emotional words. The authors used stimuli based on the most frequent words heard by psychotic patients with auditory hallucinations. When measuring activations by means of fMRI, patients as opposed to HC showed hyperactivity in the frontal lobe, temporal cortex, insula, cingulate, and amygdala.

García-Martí et al. (35) found that when hearing emotional words, high vs. low aggression violent persons with schizophrenia exhibited hyperactivation in the left hippocampus as well as in the right medial frontal cortex. The authors assume an association between the scale of aggression and certain brain regions responsible for cognitive and emotional processing. They hypothesize the alteration of neuronal circuits to favor a loss of empathic processes which could lead to aggressive behavior. These findings are partly in line with a study by Sanjuan et al. (70) where the authors found hyperactivity in the frontal lobe, temporal cortex, insula, cingulate, and amygdala when schizophrenia patients suffering from auditory hallucinations were confronted with emotional words. It remains unclear to which extent the findings by García-Martí et al. (35) are specific for violent behavior in schizophrenia.

#### Synthesis of Findings From Studies Using Emotion Induction Paradigms

**Figure 4:**
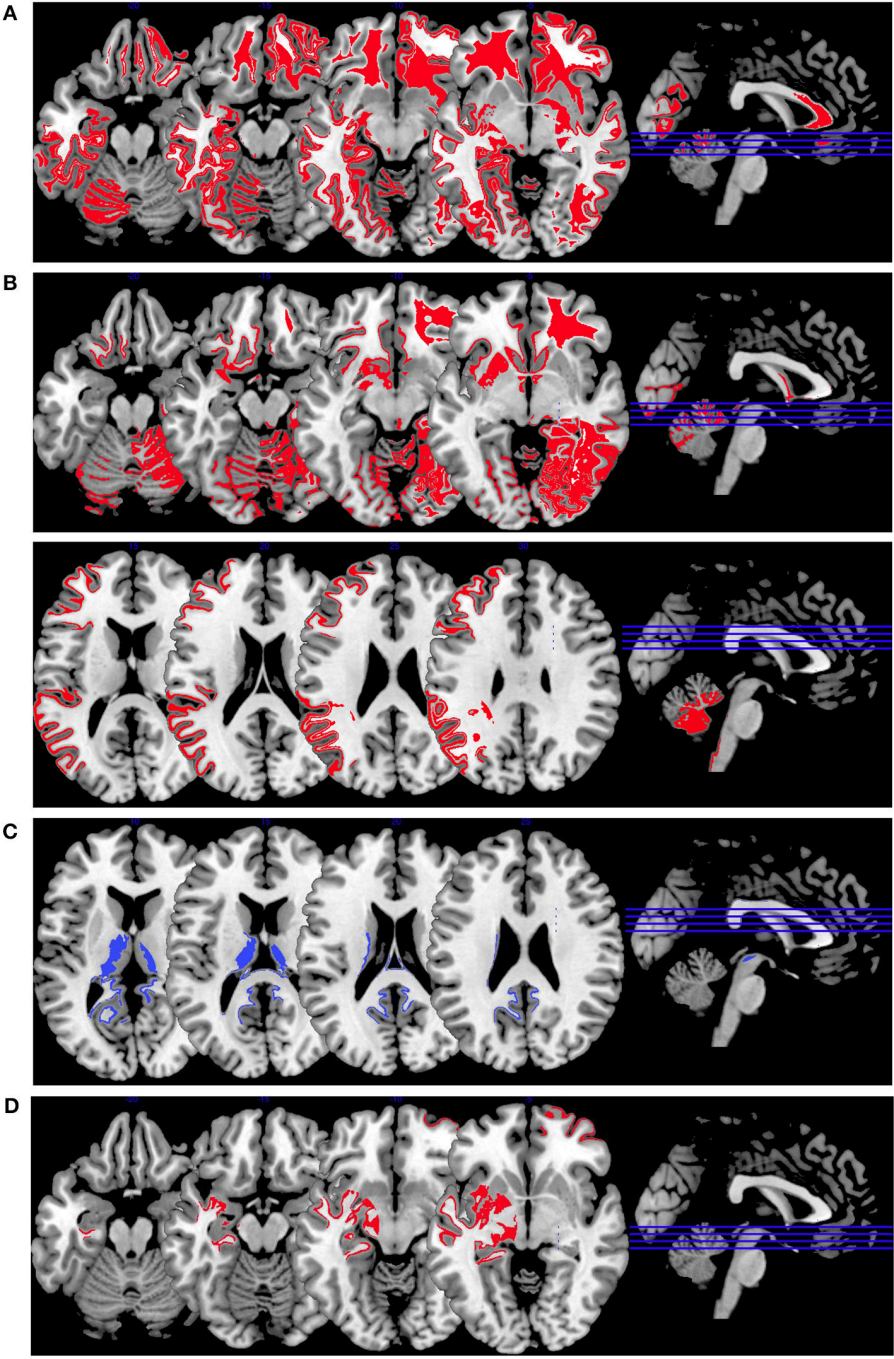
Emotion induction activations. Overview of the working memory brain activation patterns reported by the reviewed studies. Hyperactivations are shown in red, hypoactivations in blue. **(A)** shows the activation patterns in the group comparison VS vs. NVS with VS>NVS in red (shown slice numbers are 100, 110, 120, 130) in the contrasts shock anticipation phase II (last 21 s after threatening with an electric shock) vs. shock anticipation phase I (first 9 s after threat) and negative vs. neutral emotional pictures and neutral emotional pictures vs. rest, **(B)** shows the activation patterns in the group comparison VS vs. HC with VS>HC in red (shown slice numbers are 100, 110, 120, 130) in the contrasts shock anticipation vs. safe condition and in negative vs. neutral emotional pictures contrast and neutral emotional pictures vs. rest, **(C)** shows the activation patterns in the group comparison NVS vs. HC with NVS>HC in red (shown slice numbers are 170, 180, 190, 200) in the contrast negative vs. neutral emotional pictures and NVS<HC in blue (shown slice numbers are 160, 170, 180, 190) in the contrast positive vs. neutral emotional pictures, **(D)** shows the activation patterns in the group comparison NVS high vs. low aggression with NVS high>NVS low in red (shown slice numbers are 100, 110, 120, 130) in the contrast of hearing emotional words (positive and negative ones) vs. rest.

In Figure 4, we provide an overview on activation patterns during emotion induction over all reviewed studies. As with the other multislice activation pattern figures, this can be helpful to enable a synthesis of findings across the published studies. However, the emotion induction tasks used in the different studies vary considerably, and therefore this synthesis of the findings may be of limited use and must be interpreted with caution.

Figure 4A shows the group comparison of violent vs. non-violent persons with schizophrenia over all emotion induction tasks. Only hyperactivations in the right middle frontal gyrus, inferior frontal gyrus, medial prefrontal gyrus, anterior cingulate, lingual gyrus, globus pallidus, mid-cingulate, precuneus, cuneus, middle temporal gyrus, inferior temporal gyrus and in the left middle occipital gyrus, and cerebellar tuber were reported in this group comparison.

When comparing VS with HC, as shown in Figure 4B, only hyperactivations can be found. Mainly, the right hemisphere is activated (middle frontal gyrus, parahippocampal gyrus, middle occipital gyrus, anterior cingulate gyrus, cuneus, superior parietal gyrus)—hyperactivations are also seen bilaterally in the superior frontal gyrus, lingual gyrus, fusiform gyrus, superior temporal gyrus and in the left precentral gyrus, caudate nucleus, postcentral gyrus, and inferior occipital gyrus. When comparing NVS with HC (Figure 4C), there were hyperactivations in the left cerebellum and the left inferior parietal lobe and a hypoactivation in the lingual gyrus.

The activation patterns in Figure 4C differ considerably from the ones shown in the group comparison VS vs. HC in Figure 4B—we therefore suspect that emotion networks in particular may play an important role in patients with schizophrenia and aggression when in comparison with NVS. However, further studies with higher methodological quality and replication studies using comparable emotion induction paradigms are needed to test this hypothesis.

When comparing high vs. low aggressive NVS in emotion induction paradigms (Figure 4D), authors reported hyperactivation in the right superior frontal gyrus and the left hippocampus. However, it remains unclear whether these activation patterns are specific for aggression.

### Affective Theory of Mind

Affective theory of mind (ToM) refers to the ability to understand other's emotions (36) and plays an important role in aggressive behavior. Affective ToM is impaired in individuals with psychopathy—the impaired emotional responsiveness and a lack of empathy seem to be associated with ventromedial prefrontal cortex and OFC dysfunction (71). Also, the right temporoparietal junction and the cingulate cortex as well as the left supplementary motor area may be involved in affective ToM (72). Schizophrenia patients and HC do not seem to differ in brain activity during a social cognition task examining affective ToM (73).

Schiffer et al. (36) found that, when performing an affective ToM task, VS as compared to NVS hypoactivated the left ventrolateral PFC and left superior temporal sulcus at the temporoparietal junction.

#### Synthesis of Findings From Studies Using Affective Theory of Mind Paradigms

**Figure 5:**
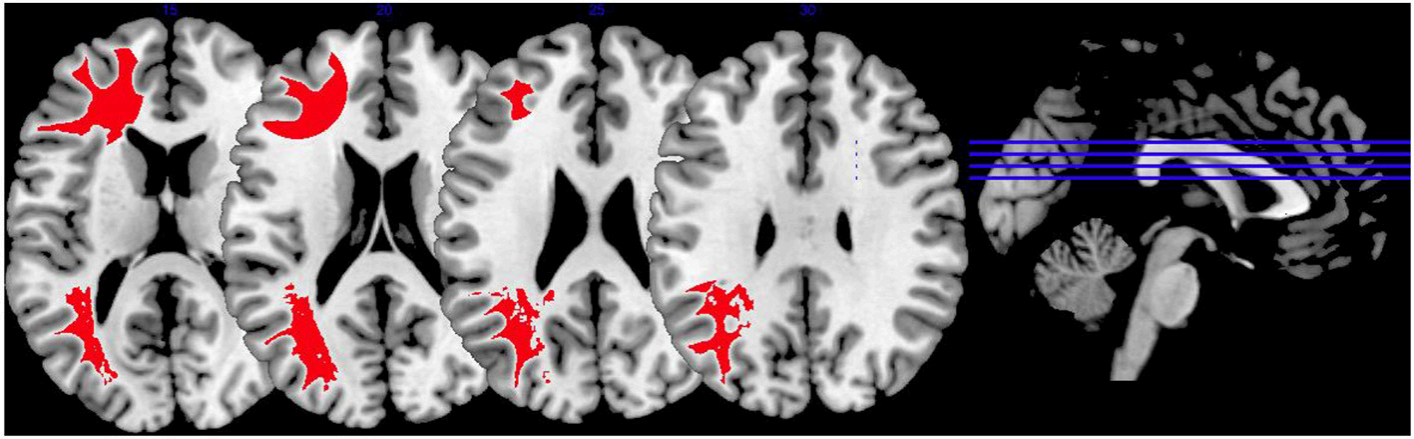
Affective theory of mind activations. Overview of the affective theory of mind brain activation patterns reported by the reviewed studies, the group comparison being VS vs. NVS in the contrast of mental state attribution vs. gender discrimination. Here, we show the hyperactivation of VS>NVS in red (shown slice numbers are 170, 180, 190, 200).

In Figure 5, we provide an overview on activation patterns generated by affective theory of mind paradigms. The only study reporting on this area was performed by Schiffer et al. (36). Hyperactivations were present in the left inferior frontal gyrus and the left superior temporal sulcus reported in the group comparison of violent as opposed to non-violent persons with schizophrenia. Here, activation of the inferior frontal gyrus is compatible with a challenge in language comprehension, while the superior temporal sulcus is known to be implicated in social perception and general theory of mind.

### Activation Patterns as Described by Other Studies

Hoptman et al. (37) conducted a resting-state fMRI study to examine amygdala/ventral prefrontal cortex connectivity in aggression and schizophrenia. Patients showed significant reductions in functional connectivity between amygdala and ventral prefrontal cortex. Lower functional connectivity was associated with higher levels of the measures *self-rated aggression, life history of aggression*, and *total arrests*. The authors conclude that amygdala and ventral prefrontal cortex functional connectivity is compromised in schizophrenia and that this compromise is associated with aggression—in other words, that reduced functional connectivity between the amygdala and the prefrontal cortex is associated with higher levels of aggression. This effect was robust when correcting for medication dosage or age and was specific to the amygdala. A previous study examining persons with schizophrenia showed reduced connectivity in the prefrontal-cerebellar and the cerebellar-thalamic limbs but enhanced connectivity in the cortico-cerebellar circuit (74). Another study found that aggressive subjects as opposed to HC did not couple amygdala-OFC during reponses to angry faces (15). The role and specificity of this decreased connectivity between amygdala and frontal brain regions remains unclear.

Spalletta et al. (38) assessed the relationship between prefrontal function and aggression in persons with schizophrenia using single photon emission tomography (SPECT). The authors compared aggressive with non-aggressive schizophrenia patients and found no difference in regional cerebral blood flow during rest. During activation with the WCST, aggressive subjects showed significantly reduced right middle and inferior prefrontal activation in comparison to the non-aggressive subjects. The authors hypothesized that prefrontal dysfunction results in the loss of intellectual flexibility impairing social skills that are essential for non-aggressive solutions. They discussed the relationship of prefrontal dysfunction to increased predisposition toward aggression and concluded that reduced prefrontal functioning could result in a loss of inhibition, which plays a role in aggressive behavior. It might be possible that patients with prefrontal dysfunction were more vulnerable to impulsive aggression due to their impaired self-control and inability to modify and inhibit behavior appropriately (38).

Wong et al. (29) conducted a positron emission tomography study in male violent offenders with schizophrenia or schizoaffective disorder and found that repetitive violent offenders showed reduced absorption of FDG at the left anterior inferior temporal cortex, while non-repetitive violent offenders (who had committed only one act of violence) had bilateral reduction of FDG uptake at both left and right anterior inferior temporal cortex. In comparison to HC, the left anterior inferior temporal cortex was significantly less activated in both patient groups. The authors conclude that anterior temporal structures are important in the control of aggression. It remains unclear whether these findings are specific to aggression, as the study has not been replicated. Wong et al. (30), in a similar study using the same cohort, reported generalized hypometabolism and discussed that these findings were non-specific because they might be the effect of mere normal aging independently of clinical circumstances.

### Limitations and Recommendations for Future Research

Both the clinical picture of schizophrenia and aggressive behavior are very heterogeneous and vary extensively. This makes examining aggression in schizophrenia-spectrum disorders using functional neuroimaging a challenge, contributes to the shortcomings of the currently published literature on this topic, and limits the explanatory power of the current systematic review. In the following, we therefore outline recommendations for future research.

#### Methodological Quality

##### Operationalization of aggression and violence

Confusion and lack of clear definitions of violence and violent behavior add to the difficulty in operationalizing aggression for study purposes (75). In most of the reviewed articles, history of violence was not specified and both type and scale of the violent acts remain unclear. Future articles should therefore clearly define and quantify the nature and extent of aggressive behavior, and adhere to common standards in the operationalization of aggression (75). The processes driving aggressive behavior remain unclear in the reviewed studies. Violence could be driven by psychotic experience (e.g., as a self-defensive response toward a perceived threat) or arise from a different developmental trajectory like for example psychopathic personality traits. Although some studies reported psychopathy checklist scores, we were unable to control for this influencing factor.

##### Predictors of violence and aggression

There is a large amount of basic and clinical research on aggression and violence in general, and on aggression and violence in persons with schizophrenia spectrum disorders, with many known predictors of these behaviors (e.g., substance use disorder, psychopathology, intelligence, psychopharmacological medication). The included studies do not sufficiently report information on these predictors. This is especially important, as co-morbid substance use disorder seems to be a very strong predictor of the risk of violence (2, 4). We recommend that future studies improve the clinical characterization of their study population and report on predictors of aggression in order to allow for moderator evaluation.

#### Sample Overlap, Lack of Replication, and Focus on Existing Paradigms

Due to the small number of available studies and considerable sample overlap, it is not yet possible to calculate effect size analyses or to conduct a meta-analysis. Future fMRI based research on the clinically important topic of aggression in psychotic disorders is therefore highly warranted. In particular, new patients should be included to avoid further sample overlap. In addition, research should focus on replication of previous findings, or at least include replication as an additional aim of study. When exploring novel study protocols and paradigms, research should seek to improve upon and enhance previous publications by focusing on similar areas. For example, focusing on the domains *working memory* and *face affect recognition* would lead to highly enhanced comparability of papers in a reasonable time period, and would allow for a better synthesis of findings.

#### Transfer of Research on Aggression and Focus on Networks

Currently, research protocols mainly focus on identifying differences on fMRI activation patterns between persons with psychotic disorders with and without aggression in an exploratory way. Although this is a viable approach, it partly neglects knowledge on aggression in healthy persons and persons with other psychiatric illnesses. Therefore, we encourage hypothesis driven research, investigating functional correlates of aggression in healthy participants and identifying functional neuronal networks of aggression. These findings should be followed-up in persons with psychotic disorders to identify common networks and differences.

#### Neglected Area: Affective Psychoses

Until now, there are no studies reporting fMRI correlates of aggression in persons with affective psychoses, although this group of persons is known to be at an increased risk for aggressive behavior. The disregard for this group of psychotic disorders severely limits the explanatory power of the literature and our understanding of aggression in psychotic disorders. We therefore recommend that future researchers include persons with affective psychoses. Although any research on affective psychosis would be valuable for the scientific community, they should also take into account the above recommendations and provide good operationalization of aggression, clinical characterization, and focus on adding knowledge with respect to existing study protocols and paradigms used in persons with non-affective psychosis.

### Conclusions

We found non-systematic functional correlates of aggression in schizophrenia. Only relatively few studies have been conducted, all using varied paradigms and often overlapping samples. Due to small sample sizes and cohort overlaps future research is highly warranted in order to gain more insight into the topic. Furthermore, no research on persons with affective psychoses has been published so far. There have been no noticeable attempts to replicate any of the observed findings in the published literature. Regarding future directions, we recommend that authors adhere to clear definitions of aggression as well as measurements of psychopathology, comorbidities, and medication. This would allow enhanced comparability of studies and moderator analyses. In addition, replication studies are needed, rather than studies focusing on new paradigms. When exploring novel study protocols and paradigms, research should seek to improve upon and enhance previous findings, and should primarily use an hypothesis driven approach, investigating functional network correlates of aggression.

## Author Contributions

CH and SW designed the study. SW conducted literature search and data extraction, and CH supervised this process and helped reach a decision in ambiguous cases. SW and CH wrote the initial draft of the paper. SW, SB, UL, R-DS, and CH made substantial contributions to data interpretation, revised the manuscript critically for important intellectual content, approved the final version of the manuscript, and agree to be accountable for all aspects of the work in ensuring that questions related to the accuracy or integrity of any part of the work are appropriately investigated and resolved.

### Conflict of Interest Statement

The authors declare that the research was conducted in the absence of any commercial or financial relationships that could be construed as a potential conflict of interest.
